# Dysregulation of the RANKL/RANK/OPG axis in thalassemia intermedia patients

**DOI:** 10.1186/s13104-018-3616-y

**Published:** 2018-07-31

**Authors:** Mahmoud A. Alfaqih, Nabil Bashir, Rami Saadeh, Yousef Khader, Musa Barqawi, Sara Alqudah

**Affiliations:** 1 Department of Physiology and Biochemistry, School of Medicine, Jordan University of Science and Technology, Irbid, 22110 Jordan; 2 Department of Family Medicine and Public Health, Jordan University of Science and Technology, Irbid, Jordan; 3 Department of Pediatrics, Princess Rahma Hospital, Irbid, Jordan

**Keywords:** Thalassemia intermedia, Osteoporosis, Bone mineral density, Receptor activator of NF-kappaB ligand, Osteoprotegerin

## Abstract

**Objective:**

Thalassemia intermedia (TI) describes a disease ranging in severity between β thalassemia major (TM) and β thalassemia trait. Osteoporosis is observed in TI and TM. The exact reason of osteoporosis in TI could be hypogonadism and/or an increase in erythropoietin (EPO) levels. The carboxy-terminal collagen cross links (CTX), a marker of bone resorption, and the N-terminal propeptide of type 1 collagen (P1NP), a marker of bone formation are serum markers of osteoporosis. The receptor activator of NF-kappaB ligand (RANKL)/receptor activator of NF-kappaB (RANK)/osteoprotegerin (OPG) axis plays an important role in metabolic bone diseases. Herein, we tested the relationship between the RANKL/RANK/OPG axis and the bone-turnover markers CTX and P1NP in TI.

**Results:**

We recruited 44 TI patients and 33 non-thalassemic controls and measured the serum levels of hemoglobin, sex steroid hormones, CTX, P1NP, RANKL and OPG. We then used a general linear model to test the association of the above variables with CTX and P1NP as outcome variables. We showed that EPO levels were the strongest predictor of CTX change (P < 0.000), followed by RANKL (P = 0.017). On the other hand, RANKL was the strongest predictor of P1NP change (P < 0.000), followed by OPG (P = 0.009) and EPO (P = 0.024).

## Introduction

Thalassemia intermedia (TI) is a term that describes β thalassemia patients in which the manifestations range in severity between β thalassemia major (TM) and β thalassemia trait [1, 2]. Among the complications associated with TI, osteoporosis is common [3]. The cause of osteoporosis is multifactorial and could be influenced by hypogonadism [4], increased erythropoietin (EPO) levels, bone marrow expansion [5], vitamin D and calcium deficiency [6]. An understanding of how the above factors determine bone loss in TI is important for the development of novel therapeutics.

Several serum markers are used in the diagnosis of osteoporosis and in monitoring response to treatment in individuals with metabolic bone diseases including thalassemia [7]. The carboxy terminal collagen cross links (CTX), a marker of bone resorption, and the N-terminal propeptide of type 1 collagen (P1NP), a marker of bone formation are commonly used [7]. Their use as bone turnover markers was endorsed by the International Osteoporosis Foundation [8].

The receptor activator of NF-kappaB ligand (RANKL)/receptor activator of NF-kappaB (RANK)/Osteoprotegerin (OPG) axis has received attention for the central role it plays in regulating bone mass density [9, 10]. RANKL is a cytokine secreted by osteoblasts in response to low calcium levels [11]. RANKL activates osteoclast differentiation from pre-osteoclasts through binding to its receptor, RANK, found on the surface of pre-osteoclasts [11]. OPG inhibits bone loss by acting as a decoy receptor to RANKL and preventing it from binding to RANK [11, 12]. Despite reports that discussed a role for the RANKL/RANK/OPG axis in determining bone loss in TM [13, 14], the contribution of this axis to bone loss in TI was not investigated. Establishing a role for the above axis in bone loss in TI will highlight its importance as a biomarker for the diagnosis of osteoporosis in this group of patients and for monitoring the response of treatment to bone anti-resorptive therapy.

## Main text

### Methods

#### Subjects

This was a comparative study carried out on TI patients attending the Thalassemic unit of Princess Rahma hospital in Irbid, Jordan. Recruitment took place between March 2015 and April 2016. Diagnosis with TI was confirmed by a pediatric hematologist based on having a hemoglobin level of 6–7 g/dl and maintaining this level without regular blood transfusions. Diabetic patients, patients with thyroid or liver disease, patients taking steroids, anticonvulsant therapies, vitamin D supplements, bisphosphonates and iron chelation therapy were excluded from both arms of the investigation. Non-thalassemic subjects were attending other clinics of the above tertiary hospital. Absence of thalassemia, hemoglobinopathies or any other blood related disorder was confirmed through hemoglobin level measurements and hemoglobin electrophoresis. Written Informed consents were obtained from all participants, or their legal guardians, according to the regulations of the Institutional Review Boards of Jordan University of Science and Technology and Princess Rahma Hospital.

#### Sample collection and processing

Two blood samples (5 ml each) were collected from each subject that met our criteria above. One sample was collected in a plain tube with a gel and clot activator (AFCO, Jordan) and another sample collected in an EDTA tube (AFCO, Jordan). Blood in plain tubes was centrifuged at 4000×*g* for 7 min, the serum was recovered from the tubes and aliquoted into Eppendorf tubes later stored at − 80 °C. Blood in EDTA tubes was used to measure hemoglobin.

#### Hemoglobin levels measurements

Hemoglobin levels measurements were performed within 15 min after collection. These measurements were performed on an HORIBA ABX Micros 60 Hematology Analyzer (Kyoto, Japan). This analyzer uses the hemiglobincyanide (HiCN) method.

#### Biochemical analysis

Serum levels of RANKL, OPG, EPO, CTX and P1NP were measured using a sandwich ELISA according to the manufacturer (Mybiosource, San Diego, CA, USA). Absorbance was determined at 450 nm on an ELx800 ELISA Reader (BioTek Instruments, Winooski, VT, USA). Serum levels of the steroid sex hormones (estrogen or testosterone) were measured using a competitive based ELISA (Mybiosource, San Diego, CA, USA). Absorbance was determined as for RANKL, OPG, EPO, CTX and P1NP.

#### Statistical analysis

The statistical package for social studies (SPSS) software (version 22, Chicago, IL, USA) was used for statistical analyses. A student t-test was used to examine if significant differences exist in age, hemoglobin, RANKL, OPG, RANKL/OPG ratio, EPO, sex steroid hormones, CTX, P1NP between thalassemic and non-thalassemic subjects. A general linear model was used to test the effect EPO, Estrogen/Testosterone, OPG, RANKL on the two outcome variables (CTX and P1NP). The null hypothesis was rejected if P-value < 0.05.

## Results

Forty-four patients with TI and 39 non-thalassemic individuals met our criteria. No significant differences existed in gender distribution and in age between the non-thalassemic and thalassemic subjects (Table 1). The biochemical profile showed that thalassemic subjects had higher levels of serum erythropoietin (EPO) (638.4 ± 272.3 vs. 137.7 ± 81.5 ng/ml, P < 0.0001), RANKL (4980.9 ± 1313.6 vs. 2621.9 ± 578.9 pg/ml, P < 0.0001) and CTX (459.2 ± 120.1 vs. 185.9 ± 53.6 pg/ml, P < 0.0001). On the other hand, the thalassemic subjects had lower levels of OPG (3370.0 ± 1142.4 vs. 3931.2 ± 479.8 pg/ml, P = 0.004), estrogen/testosterone (0.63 ± 0.44 vs. 2.7 ± 0.9 pg/ml, P < 0.0001) and P1NP (64.4 ± 52 vs.269.4 ± 127.7 pg/ml, P < 0.0001) (Table 1). Noteworthy, all the thalassemic subjects had changes in both sex steroid hormone and EPO levels. None of the patients recruited to this study had changes in only one of the above two parameters (data not shown).

**Table 1:** Baseline characteristics of thalassemic and non-thlalassemic subjects

Variable	Non-thalassemic	Thalassemic	P-value^a^
Gender (n)			
Females	16	15	0.515
Males	23	29	
Total	39	44	
Age (mean ± SD)^b^	24.59 ± 8.165	23.41 ±7.916	0.506
Hb, g/dl (mean ± SD)^b^	14.5 ± 1.42	7.8 ± 0.71	< 0.0001
EPO, ng/ml (mean ± SD)^b^	137.7 ± 81.5	638.4 ± 272.3	< 0.0001
Estrogen or testosterone, ng/ml (Mean ± SD)^b^	2.7 ± 0.9	0.63 ± 0.44	< 0.0001
OPG, pg/ml (mean ± SD)^b^	3931.2 ± 479.8	3370 ± 1142.4	0.004
RANKL, pg/ml (mean ± SD)^b^	2621.9 ± 578.9	4980.9 ± 1313.6	< 0.0001
RANKL/OPG ratio (mean ± SD)^b^	0.7 ± 0.22	1.66 ± 0.85	< 0.0001
P1NP, pg/ml (mean ± SD)^b^	269.4 ± 127.7	64.4 ± 52	< 0.0001
CTX, pg/ml (mean ± SD)^b^	185.9 ± 53.6	459.2 ± 120.1	< 0.0001

*Hb* hemoglobin, *EPO* erythropoietin, *OPG* osteoprotegerin, *RANKL* receptor activator of NF-kappaB ligand, *RANK* receptor activator of NF-kappaB, *P1NP* N-terminal propeptide of type 1 collagen, *CTX* carboxy terminal collagen cross links

^a^P-values were calculated by the Student’s t-test

^b^Data are presented as the mean ± standard deviation

Given that RANKL and OPG levels (i.e.: components of the RANKL/RANK/OPG axis) were different in thalassemic compared to non-thalassemic subjects, we then tested the association of RANKL and OPG with the bone-turnover markers CTX and P1NP. To this end, we used a general linear model with CTX and P1NP as outcome variables. RANKL, OPG, EPO and estrogen/testosterone were predictor variables. Our analysis showed that EPO was the strongest predictor of CTX (P < 0.000), followed by RANKL (P = 0.017) and then by estrogen/testosterone (P = 0.029). OPG was not a significant predictor of CTX change in this analysis (Table 2).

**Table 2:** Prediction of changes in CTX and P1NP using a general linear model

Variables	CTX, pg/ml (330.759 ± 166.5)		P1NP, pg/ml (160.703 ± 139.9)	
	P-value^a^	Partial Eta squared	P-value^a^	Partial Eta squared
EPO	< 0.0001	0.152	0.024	0.064
Estrogen or testosterone	0.029	0.060	0.816	0.001
OPG	0.335	0.012	0.009	0.083
RANKL	0.017	0.071	< 0.0001	0.194

*CTX* carboxy terminal collagen cross links, *P1NP* N-terminal propeptide of type 1 collagen, *EPO* erythropoietin, *OPG* osteoprotegerin, *RANKL* receptor activator of NF-kappaB ligand

^a^P-values were calculated by a general linear model with CTX and P1NP as outcome variables

On the other hand, RANKL was the strongest predictor of P1NP change (P < 0.000), followed by OPG (P = 0.009) and EPO (P = 0.024), while estrogen/testosterone was not a significant predictor of P1NP1 change. RANKL and EPO were the only predictors associated with both CTX and P1NP in the model with EPO explaining 15% of the variation of CTX (Partial Eta squared = 0.152) while RANKL explaining 19.4% of the variation of P1NP (Partial Eta squared = 0.194) (Table 2).

## Discussion

The above findings provide evidence on the presence of changes in the levels of several serum markers that could indicate bone loss in TI patients. Changes in the serum levels of these markers are used in other disease conditions, including TM, to establish the presence of osteoporosis. Although premature, these changes are discussed in the context of how they could mechanistically contribute to osteoporosis in TI based on previous reports that discuss the contribution of these markers to bone loss in other disease conditions.

Notable was our finding that the serum levels of the sex steroid hormones estrogen or testosterone were significantly lower in TI patients compared to non-thalassemic subjects. The cause of the hypogonadism observed in thalassemia patients is multifactorial and could be related to increased iron stores in gonadotrophic or pituitary tissues [15, 16]; a condition that predisposes these tissues to peroxidative damage caused by the generation of free oxygen radicals through fenton reactions [17]. The observed hypogonadism may be involved in the disruption of bone homeostasis in TI. Several reports showed that estrogen, through its interaction with estrogen receptor α, increases the transcript and protein levels of OPG [18]. Moreover, estrogen was reported to decrease the expression levels of RANKL [19]. The above changes are predicted to cause disruption of bone remodeling in TI with a resulting increase in the rate of bone resorption and a decrease in bone formation; pathophysiological changes that are consistent with our findings that CTX (a marker of bone resorption) serum levels are significantly increased in TI patients while P1NP (a marker of bone formation) levels are significantly reduced. Whether a reduction in sex steroid hormone levels is the driving force for bone loss in TI patients remains to be determined, nonetheless our findings support the use of selective estrogen receptor modulators (SERMs) for the treatment/prevention of bone loss in TI [20].

We also found that RANKL serum levels were significantly higher in TI patients compared to the non-thalassemic controls, while OPG levels were significantly lower in TI patients. Furthermore, we found that RANKL was a significant predictor of the levels of CTX and P1NP while OPG was a significant predictor of P1NP serum levels. These findings support that bone remodeling in TI patients may be dysregulated because of an imbalance between the rate of bone formation and bone resorption. It appears that there is a decrease in the rate of bone formation accompanied with an increase in the rate of bone resorption with a net result of bone loss in TI. We also demonstrated that RANKL, OPG or RANKL/OPG ratio could serve as potential serum bone-turnover markers in TI.

In addition to the biochemical changes above, we found that EPO levels increased in thalassemic patients. We also found that EPO serum levels were a significant predictor of changes in both CTX and P1NP levels. The increase we observed in EPO levels is presumably reflective of an increase in tissue hypoxia resulting from the anemia that accompanies thalassemia. In this case, EPO stimulates the bone marrow to produce more red blood cells to compensate for the low hemoglobin that accompanies thalassemia. An increase in EPO serum levels may also contribute to the bone loss observed in TI where erythroid hyperplasia, caused by increased EPO levels, may cause cortical thinning and an increase in bone fragility [21].

It is well established that the clinical/complication profile of TI differs from TM [22]. For example, unlike patients diagnosed with TM, TI patients present with the disease in later childhood or early adulthood and do not require regular blood transfusion to manage their anemia [22, 23]. Distinction needs to be made between TI and TM patients to spare TI patients from the side effects associated with the often unnecessary blood transfusions [24]. Distinction is purely made on clinical basis, although genotype/phenotype associations in TI were described [25]. These associations support the feasibility of finding genetic/biochemical markers that distinguish between the two disease states. Given our findings that the blood levels of the sex steroid hormones, EPO, OPG and RANKL change in TI, it remains to be determined whether measurement of these markers could help distinguish between TI and TM. However, this requires the recruitment of both TM and TI patients followed by the measurement of the above markers; a future direction of our research group.

In conclusion, up to our knowledge, our research team is the first to report changes in the serum levels sex steroid hormones, EPO, OPG and RANKL in TI patients compared to non-thalassemic controls. The translational relevance of these findings to the treatment of osteoporosis in TI and the distinction between TI and TM still needs to be determined.

## Limitations

The sample size may limit the statistical power of this investigation and further validation studies on a larger group of patients is warranted to confirm our findings. Additionally, we failed to include a direct measurement of bone density using dual-energy X-ray absorptiometry (DEXA) [26]. Instead, we relied on indirect evaluation of bone density inferred by measurements of the bone turnover markers CTX and P1NP. The lack of such data prevented us from correlating the serum levels of the sex steroid hormones or EPO with the severity of the disease.

## Abbreviations

TI: thalassemia intermedia
TM: β thalassemia major
EPO: erythropoietin
CTX: carboxy-terminal collagen cross links
P1NP: N-terminal propeptide of type 1 collagen
RANKL: receptor activator of NF-kappaB ligand
RANK: receptor activator of NF-kappaB
OPG: Osteoprotegerin
ELISA: enzyme-linked immunosorbent assay
SPSS: statistical package for social studies
SERM: selective estrogen receptor modulators
DEXA: dual-energy X-ray absorptiometry
